# How cancer patients get fake cancer information: From TV to YouTube, a qualitative study focusing on fenbendazole scandle

**DOI:** 10.3389/fonc.2022.942045

**Published:** 2022-10-28

**Authors:** Jee Hyun Kim, Kyoung Hee Oh, Hye Young Shin, Jae Kwan Jun

**Affiliations:** ^1^ National Cancer Control Institute, National Cancer Center, Goyang, South Korea; ^2^ Department of Nursing, Gangseo University, Seoul, South Korea; ^3^ Graduate School of Cancer Science and Policy, National Cancer Center, Goyang, South Korea

**Keywords:** complementary therapies (MeSH), lung neoplasms, YouTube, mass media, fenbendazole, focus groups, health communication

## Abstract

**Background:**

Korean society has faced challenges in communicating with cancer patients about false information related to complementary alternative medicine. As the situation has become severe with the 2020 fenbendazole scandal, the demand for reliable information from health authorities has increased.

**Objectives:**

This study aimed to examine patients’ acquisition patterns and perception of false information by presenting empirical evidence to help health authorities enable effective preemptive responses in the cancer communication context.

**Method:**

We conducted a focus group interview with 21 lung cancer patients who were informed about fenbendazole based on a semi-structured questionnaire with three categories: 1) acquisition channel of the general cancer information and the false information, 2) quality of obtained information, and 3) perception toward it. The interviewees, comprising 13 men and eight women, were aged 50 or older. Participants’ current stages of cancer were stages one, three, and four and there were seven people in each stage.

**Results:**

1) Acquisition channel: Participants had their first encounter with false information through the TV, while the channels to obtain general cancer information were through Internet communities or portal sites. YouTube was a second channel to actively search for information regardless of the information type. 2) Information quality: participants had only fragmented information through media. 3) Perception: Most patients had a negative attitude toward complementary and alternative medicine information such as fenbendazole. They perceive that it needs to be verified by experts and filtered according to their arbitrary criteria. They had vague expectations based on a hope for “what if” at the same time.

**Conclusions:**

Despite the complex media environment, traditional or legacy media is an important channel to encounter information. YouTube is independent of other media as an “active” information-seeking channel. Patients required the appropriate intervention of experts and governments because they perceived that they had obtained irrational and unreliable information from the media. Suggestions are made about how health authorities can construct an effective communication system focusing on the user to prevent patients from getting false cancer information.

## 1 Introduction

The “fenbendazole scandal” is associated with bitter memories in Korean society as official health communicators could not control or filter false cancer information. As the information was revealed to be wrong after a long controversy that lasted more than a year, the need for a national-level response has been raised. Hence, this scandal was considered a representative example of the problem of using complementary and alternative medicine (CAM) for cancer patients during the 2020 parliamentary audit of the Ministry of Health and Welfare.

The fenbendazole scandal was an incident wherein false information that fenbendazole, an anthelmintic used to treat various parasites in dogs, cured terminal lung cancer spread among patients. It started with the claim of American cancer patient, Joe Tippens, but rather became sensational in South Korea. It caused national confusion and led to fenbendazole being sold out at pharmacies across the country in South Korea. Contrary to what the people know, however, Joe Tippens was a participant in the Kitruda clinical trial at the MD Anderson Cancer Center, and his improvement was likely to be the effect of immuno-cancer drugs. So, at the beginning of the issue, health authorities and experts in South Korea warned about side effects through press releases on September 23, 2019, but cancer patients ignored them. Instead, people were interested in a celebrity, a famous comedian in Korea, taking fenbendazole. The false information continued to spread until the celebrity declared that “it is ineffective.” (The celebrity announced his intention to take fenbendazole on Social Networking Service (SNS) on September 24, 2019, while suffering from lung cancer. In September 2020, he mentioned that he would not take fenbendazole or recommend it. He eventually died in December 2021). Why was the warning given by the health authorities ineffective? How should the government respond if false information is spread in the cancer information market again? Answering these questions remains a significant challenge related to cancer communication in Korea. Hence, there is a need for an academic approach.

The fenbendazole scandal was considered an issue wherein false information related to CAM confused cancer patients in Korea (1). The academic definition states CAM is “an unusual treatment, lacks scientific evidence, and is personally used for cancer treatment or health (2, 3).” Cancer patients are known to use various CAM throughout the treatment and recovery process (4–9). False information that fenbendazole is effective in curing terminal cancer shook the cancer information market, despite warnings from experts and the absence of clinical research. This could be because it had been accepted as a CAM, which was familiar to them. Previous studies about CAM can be categorized as follows: (1) the reasons for using CAM (10–13), (2) demographic characteristics of people using CAM (9, 14–16), and (3) the types of CAM mainly used (7, 14, 17–19). Some studies analyzed information sources and argued that CAM information is mainly obtained from family members, acquaintances, mass media, and patients (16, 20, 21). However, there are few empirical studies on where and how cancer patients obtain CAM information considering the complex media environment with a massive amount of Internet materials and OTT-based channels. Specifically, a few studies have examined the process of acquiring false information related to CAM and their perception of it.

In Korea, the number of cancer patients exceeded two million (22, 23). Accurate and reliable cancer information is essential for cancer patients to continuously manage this chronic disease independently (24–26). For cancer communication to focus on the effective delivery of cancer information, a comprehensive understanding of the context of its acquisition needs to be preceded. Based on situational and academic discussions, this study aimed to examine the context of acquiring false information on cancer treatment by cancer patients, focusing on the “fenbendazole scandal,” and false CAM information.

This study extracted qualitative data from lung cancer patients through focus group interviews (FGI). The aims of this study are threefold: (1) to identify how cancer patients acquire fenbendazole information compared to general cancer information, (2) to examine the quality of the information obtained by various channels, and (3) to identify cancer patients’ perception of it.

## 2 Methods

This study conducted focus group interviews with lung cancer patients. A qualitative research method provides practical data that can best reveal participants’ experiences, specific cases, and contextual experiences (27, 28). Hence, it would be a suitable method to conduct a detailed examination of the contextual process of acquiring information on fenbendazole and their perception of it.

### 2.1 Participants and focus group interview procedure

A total of four focus groups with five to six lung cancer patients were interviewed. A total of 21 lung cancer patients (13 men and eight women) were randomly assigned. The interviewees were lung cancer patients being treated at a large hospital. Participation was voluntary and there were no additional inclusion or exclusion criteria except for the perception of fenbendazole among patients with lung cancer. A moderator with experience with various FGI for cancer patients was responsible for these interviews. These were held from December 7 to 8, 2020, and lasted for about 1.5 h. Before FGI started, the participants were informed again about the topics and the moderator’s role. A semi-structured questionnaire was used and its contents were divided into three categories: (1) the information acquisition process of fenbendazole and general cancer information, (2) the quality of obtained information, and (3) the perceptions of the information among cancer patients.

### 2.2 Analysis

The interviews were tape-recorded, and the discussion materials were transcribed by a professional service. To ensure anonymity in the transcribing process of the recorded version, each participant was arbitrarily classified as an alphabet (e.g., A, B, C….). Researchers confirmed no omission or inaccuracy in the transcribed data and analyzed it using an established method (29). The first author read all the transcripts several times, wrote case summaries based on the main questions and what the cancer patients had expressed during the interviews, and synthesized information from different parts of the transcripts. These were reviewed and discussed by three researchers. Then, the information was categorized across the case to create sub-categories based on analysis. All researchers read and repetitively analyzed the texts to find commonalities and differences among the participants while adding more details and avoiding data distortion. This study was approved by the National Cancer Center Institutional Review Board in Korea (approval number: NCC2022-0001).

## 3 Results

The interviewees’ ages ranged from 56 to 75 years, and the average age was 66.6 years Participants were first diagnosed with lung cancer three months to five years ago. Participants’ current stages of cancer were stages one, three, and four (**Table 1**). According to this study’s aim to examine where cancer patients get false and general cancer information, the quality of the information, and how they perceive it, we have divided the results into three categories as follows: (1) information acquisition process, (2) quality of information, and (3) perception and attitude toward information.

**Table 1:** Study participants’ general characteristics.

Characteristics	N	%
**Sex**		
Men	13	61.9
Women	8	38.1
**Age**		
55−59	7	33.3
60−64	3	14.3
65−69	7	33.3
Over 70	4	19.1
**Education**		
Middle-school	2	9.5
High-school	3	14.3
University degree	14	66.7
Graduate degree	2	9.5
**Stage of cancer at diagnosis **		
1	7	33.3
2	–	
3	7	33.3
4	7	33.3
**the years since diagnosed**		
Less than a year	2	9.5
1-2 years	9	42.9
3-4 years	7	33.3
Over 5 years	3	14.3

### 3.1 Category 1. Information acquisition process

This category has two separate research questions. One, the process of acquiring fenbendazole information, and two, the process of acquiring general cancer information daily.

#### 3.1.1 The process of acquiring fenbendazole information

Most cancer patients first encountered fenbendazole through TV reports on celebrities taking fenbendazole (A, C, D, E G, H, K, M, O, Q, and T). G explained, “*I saw fenbendazole on TV. Kim Chul-min said he got better after taking it.* “Cancer patients explained that, even if they do not see it directly, once it is reported on TV, it seems to have a significant influence because family members or acquaintances can obtain and share the information. Specifically, E said, “*Since we usually watch information programs, including TV news, we mainly get information through them. Furthermore, my family, friends, or acquaintances can come across the information on TV … For example, my son sees the information first and passes it on to me.”*

Along with TV, acquaintances or family members were also the first channels through which patients got the information (B, J, L, P, and S). B and L explained that *they had heard information about fenbendazole from an acquaintance while they were in the nursing home. The acquaintance was Kim Chul-min (they met by chance)*. Some people first encountered it on the Internet or YouTube, but the percentage was the lowest (C, N, and U). N said *he had first encountered the information at an Internet cafe*, and U said that *he had seen it on YouTube and TV news*.

After encountering fenbendazole, most participants cited YouTube as the channel through which they obtained additional information (A, C, I, J, K, N, R, and S). YouTube is directly accessed through its application and not through a search engine. K’s answer could be a representative example: *I first saw it on TV. It was a news report. Then I searched YouTube and saw a lot of information on it.* However, patients did not search for additional information only through YouTube. Participants said they had also searched on the Internet portal sites. They clicked on the various sources such as news articles, Q&A, or blogs from portal sites, simultaneously and randomly. R mentioned: *I looked it up YouTube a lot, but I also searched the Internet including blogs, news articles, and Q&A, and checked if it was effective. I read about many patients’ experiences.*

#### 3.1.2 The process of acquiring general cancer information

The main channels used by cancer patients to obtain cancer information daily are the Internet (A, B, C, G, H, J, K, L, O, P, and U) and acquaintances (D, M, Q, S, and T). Specifically, information was mainly obtained from Internet communities (A, B, C, H, J, and U). Internet information channels, excluding communities, were portal site searches (G, L, and O). Various information sources from Internet portal sites like blogs, Q&A, or news articles are used regardless of the cancer stage. However, Internet cafes are usually used in the early stages of cancer diagnosis, and their use decreases over time. This is because they mainly include basic information on specific cancers or hospitals during the early stages and there are many commercial aspects and extreme cases. They explained that as cancer continued, the information that they needed was scarcely available (B, K, and P). B said in detail about his experience using an Internet café after being diagnosed with cancer: “*After being diagnosed, I searched all day, only to find one or two cafes and unconditionally registered as a member. But now, I am in the state of taking a CT scan without any special treatment. The information I need is no longer in the cafe. So the use of café information is sparse.* K pointed out that there are many extreme cases in Internet cafes: *For example, there are people who have relapsed after surgery in stage 1 in the cafe. However, in reality, the probability of recurrence in stage 1 is only 20%.* However, most participants responded that they read cancer information about once a week or once every two weeks on average, indicating that patients generally do not search for cancer information daily or frequently (A, B, C, D, F, I, L, M, O, Q, and S).

**Figure 1:**
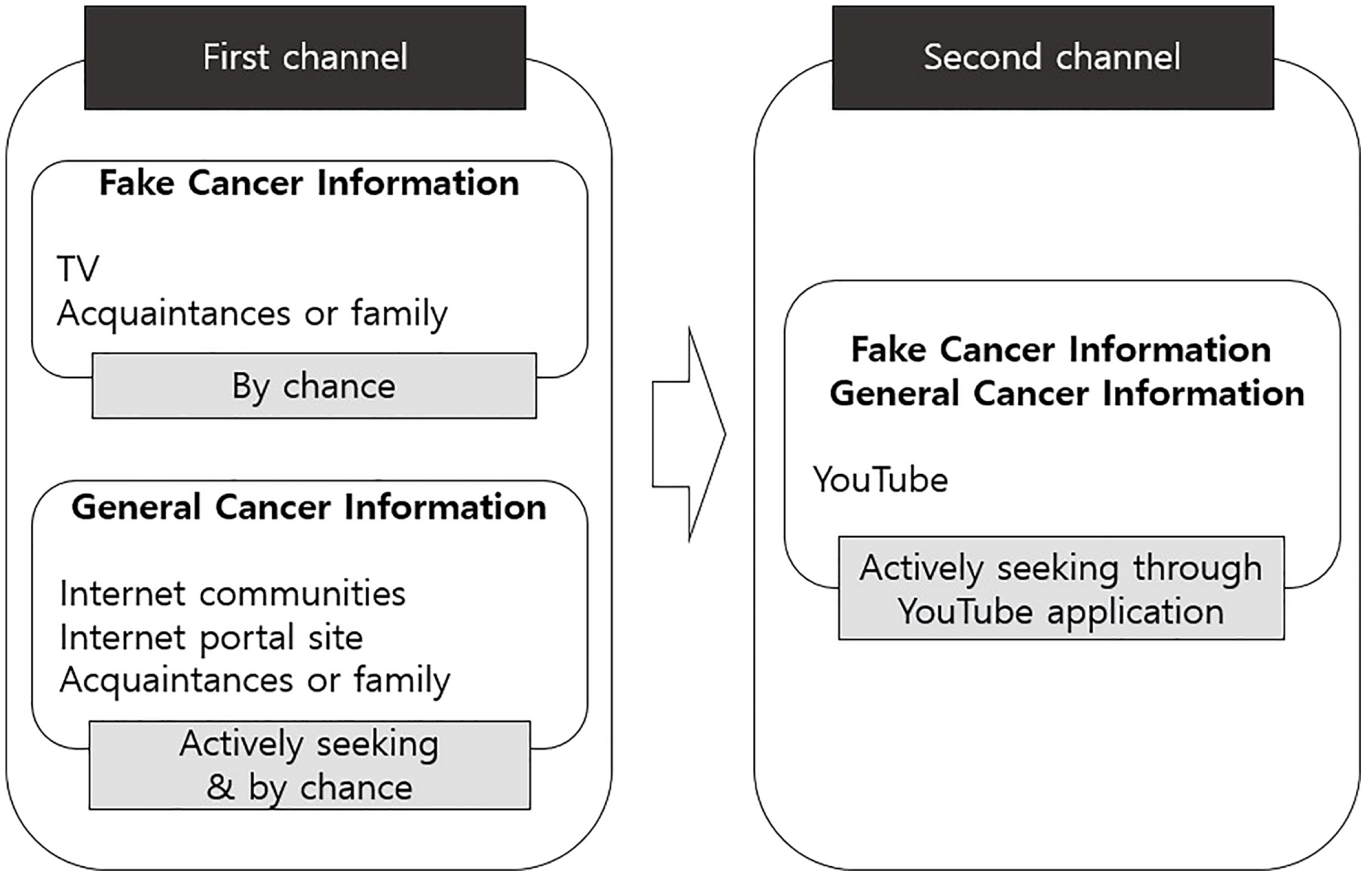
The process of acquiring cancer information.

YouTube was rarely mentioned as the first acquisition channel for general cancer information. However, it functioned as a secondary information-seeking channel. For example, participants first obtained information from the internet patient cafes and then used YouTube to find out more. N said: *I generally watch information in Internet cafes and, if it interests me, I search on YouTube. I use YouTube to find additional things.* The aforementioned results are visualized in **Figure 1**.

### 3.2 Category 2. Quality offenbendazole information

The quality of information obtained was analyzed by asking how accurate information participants knew about fenbendazole issue. So, this category consisted of questions related to two key figures related to fenbendazole. One is about Jo Tippens, who first claimed the effectiveness of fenbendazole, and the other is about the celebrity who made fenbendazole a social issue in Korea.

#### 3.2.1 Fragmented information obtained by the media

Most people who acquired fenbendazole information through media channels such as TV and the Internet knew only a small part of it. To examine its accuracy, interviewees were asked if they knew Kim Cheol-min, who first became an issue for taking fenbendazole in Korea, had been taking fenbendazole and continued chemotherapy at the hospital simultaneously. Participants mentioned that they did not know that he was undergoing chemotherapy (A, B, F, G, H, I, M, O, J, Q, S, and T). S said, “*I thought Kim Cheol-min only took fenbendazole. The title of the news article said he only took dog anthelmintics. It did not mention that he was taking anticancer drugs.”*

Additionally, few people knew that Joe Tippens, who first claimed the effect of fenbendazole, was undergoing other treatments. If patients tried to find more information through YouTube and did not access the original data, the obtained information was fragmented and incomplete as processed by other media channels. Only two participants, K and U, responded that *they directly looked up Joe Tippens’ video on YouTube after acquiring information on fenbendazole through media and knew about the fact. They actively cross-checked information through the original videos on YouTube.*

### 3 Category 3. The perception and attitude toward CAM information

#### 3.3.1 Negative attitude: 1) Needs to be verified by experts

Patients have difficulty accessing the original data or accurate information because cancer information belongs to a specialized area of medical facts. Participants mentioned that although cancer information should be medically verified, there is exaggerated information, and commercials are not verified on TV or the Internet (B, C, E, I, K, P, R, S, T, and U). C said, “*I know that the process of verifying one drug is difficult and time-consuming. Regarding fenbendazole, it has not been verified. It is difficult to know the accuracy of the information unless experts verify it.”*

Despite the growing number of channels to obtain information, patients feel that there is a lack of cancer information as CAM information generally does not provide scientific evidence and there is little information that they need (A, B, D, G, K, L, N, O, and P). Participants explained that the information on how to take care of themselves, especially after surgery or chemotherapy, when staying at home for self-care and boosting immunity is not sufficient (A-U). T said, “*The hospital provides treatment, but after that, most of us are at home. Doctors do not talk about how to manage at home and I cannot get reliable information through the media.”*

All participants hoped that experts would come forward with cancer information (A-U). They mentioned it would be good for medical staff or officials to sort out this confusing situation because only experts can verify and deliver reliable information. B explained, “*We know that cancer gets better or worse depending on how doctors treat it. But, how should I take care of my body so that it does not happen again? We need this information. I wish doctors or other hospital staff could teach me.*” Most people mentioned the experts’ intervention for CAM information related to dietary therapy. J said that *it would be better if the doctor gave an example of the foods that are good for my body and which should be avoided rather than saying, “Eat well.”* P said, “*If you look on the Internet, some say you should eat red ginseng, and some say you should not. What should I do to boost my immunity? I need such information, and I hope the experts will tell me.*”

#### 3.3.2 Negative attitude: 2) Needs to be filtered by self

Patients always try to check the information’s accuracy using the available channels, but since it is difficult, they eventually rely on arbitrary and subjective criteria. All participants said *they filtered the information using personal judgment* (A-U). Participants specifically explained that *the judgment has to be made by themselves because support is not available.* They added that *if they make judgments based on common sense and search the Internet, they can create their own criteria* (B, E, F, and R). They mentioned that the accuracy of the information is learned by applying it directly to their bodies (B, E, G, K, L, M, N, O, P, and R). P explained*, “It is difficult to explain exactly, but I can feel whether the information is an exaggerated advertisement. When I think that the information is true, I apply it to my body. Then I can check if it works.”*

#### 3.3.3 Vague expectations for CAM

When cancer patients first encountered the issue of fenbendazole, most of them thought it was difficult to trust as it was “unverified” (A-U). U and C explained that *they do not believe it completely as too many things have not been verified*. However, as aforementioned, *in the media, there is not much reliable CAM information because experts such as doctors rarely provide direct information on medicines such as fenbendazole.* However, people who have been exposed to information through various channels have expectations of “what if”. At this time, what serves as strong “evidence” for patients, despite no scientific evidence, is the experiences of those who have the same disease.

The credibility of the information on fenbendazole was low; however, the expectation made cancer patients conduct self-assessments (B, C, E, I, K, N, P, R, S, and T). As a typical and representative example, K said, “*It does not make sense to say that only fenbendazole made them better. They must have done many things, including chemotherapy. But I have tried it. No one knows if it will work … But now, I stopped taking it because of my liver.* C also mentioned the positive attitude due to such expectations. He said, “*Cancer patients have certain expectations. We all know the feeling of desperation. Even if you think it makes no sense rationally, when I hear a story like someone ate something and recovered … it sparks the hope for ‘what if’. Even with the slightest hope like this, I want to try it.* Specifically, all participants said that, while seemingly irrational like fenbendazole, the more dramatic the therapeutic effect, the more terminally ill patients would take it (A-U). “*I am dying of cancer. What do I do? You can do it all. If anthelmintics used for dogs is good for terminal cancer, who does not want to take them? Everyone will want to, even if there is no scientific basis. No one can tell them whether they are right or wrong rationally.”* (I) For that reason, in general, CAM such as dietary supplementation, health supplements, and Chinese medicine, were routinely performed by cancer patients without any objection (17 out of 20). The aforementioned results are summarized in **Table 2**.

**Table 2:** Categories and sub-categories with coding example.

Categories	Sub-categories	Coding example
Category 1 Information acquisition channel	Information on fenbendazole obtained by: **Mass media,** **Internet portal** **YouTube** **Acquaintance/family**	*I first saw it on a TV news report. Then I searched YouTube and read a lot about it. *I read about fenbendazole at an Internet café. *I saw it on YouTube and TV news. *I heard it from an acquaintance while I was in the nursing home. *My family told me the fenbendazole after having seen it from TV.
	General cancer information obtained by: **Internet portal** **Acquaintance/family** **YouTube**	*Generally, I search the Internet, regardless of the articles, blogs, and Q&A *I heard it from my daughter * While surfing the Internet, if I come across anything interesting, I search YouTube. I use YouTube to find additional things
Category 2 Quality of information	Fragmented information obtained by media	*I thought Kim Cheol-min only took fenbendazole. The title of the news article said he only took dog anthelmintics. It did not report that he was taking anticancer drugs.
Category 3 Perception and attitude	Negative attitude: **Need to be verified by experts** **Needs to be filtered by self**	*Regarding fenbendazole, it has not been verified. It is difficult for us to know whether the information is true unless experts verify it. *It is difficult to explain exactly, but I can feel whether the information is an exaggerated advertisement. I have to filter it. When I think that the information is true, I apply it to my body. Then I can see if it really works.
	Vague expectation	*No one knows if it will really work *When I hear a story like someone ate something and recovered … it arises a hope for “what if”. Even with the slightest hope like this, I want to try it.

## 4 Discussion

This study aimed to explore where cancer patients as information consumers get socially controversial cancer information compared to general cancer information in the complex media environment, quality of the information, and their perspectives about it. According to the research purpose to obtain empirical and contextual information through FGI of cancer patients, there are some interesting findings through qualitative studies.

The first is about the process of acquiring information from cancer patients. This study’s results revealed the following: 1) cancer patients use various media and channels step-by-step to obtain cancer information, and 2) the process of acquiring socially issued cancer information and general cancer information daily is different. Daily cancer information acquisition was mainly through the Internet followed by YouTube, and information that received social attention, such as on fenbendazole, was obtained through TV followed by YouTube. Based on this, we can conclude that the acquisition of general cancer information showed the same pattern as previous research (30), but the fenbendazole information revealed a similar pattern to the rumor diffusion model (31, 32), which is different from the path on a daily basis. Specifically, the first fenbendazole information is obtained passively, mainly through TV. Sharing information or opinions among users has become common on the Internet community or social media; however, mass media plays an important role (31) as it spreads rumors even to those who do not know them (those who are “ignorant”). Fenbendazole scandal is similar to the characteristics of rumors as it lacks scientific evidence, is controversial, and requires fact-checking (33). These findings suggest that policymakers should consider the important role of mass media in establishing a response system to false information that becomes a big issue despite inaccurate scientific evidence and causes social confusion.

The aforementioned results also show that deeper discussions on YouTube as a meaningful information channel need to be conducted academically and practically. Cancer patients did not choose YouTube as the first channel to obtain fenbendazole information, but it was an independent channel that most patients used to find additional information. Previous studies argued that consumers use YouTube channels as a kind of complementary media to explore the truthfulness of a specific issue (34). However, YouTube is an important channel in the active information acquisition process of cancer information. This study’s results revealed that YouTube is functioning as a separate information channel to search for people’s experiences and various opinions apart from Internet portal sites (Participants mentioned that they searched Internet portal sites and YouTube separately to get more information). Some scholars who recently analyzed the media ecosystem used the term “YouTube journalism” and regarded its political and current affairs channel as an expansion and reinforced version of existing journalism (35–37). They pointed out that YouTube’s influence is growing wide enough to match legacy media and portal sites, arguing that the former’s role will gradually reduce. However, regarding cancer information, diverse media, including TV and various platforms, show a pattern of coexistence in the process of acquiring cancer information with their respective roles. Hence, policymakers need to establish an organic connection system with mass media and YouTube and how to actively use YouTube in responding to false cancer information.

The second finding is about the quality of the information obtained through the media. It is related to a critical discussion on the journalistic function of TV as the first channel for false information acquisition channel. As mass media has the power to spread information to even those who do not know about the issue, the accuracy and sufficiency of media information needs to be seriously discussed. Scholars argue that considering its influence, the mass media should report accurate information in a balanced manner to help the general public make correct judgments on specific issues. The results of this study raise fundamental questions about whether the media has reported accurate and evidence-based information from various perspectives. Therefore, we suggest that a detailed content analysis of the cancer information delivered by mass media needs to be conducted. It will provide various implications for the reporting pattern of cancer information in health journalism. For example, it seems that we can seriously discuss various journalistic values such as information accuracy, diversity of perspectives, and depth of the news content, which is the basic value of journalism (38, 39). Meanwhile, despite the increasing acquisition of information through YouTube and improving public belief in YouTube channels (40), there are few empirical analyses of how and by whom cancer information is produced and distributed on YouTube. As the people of Korea use YouTube as an information acquisition channel approximately 20% more than other countries (41), it would be meaningful to analyze the production and distribution of cancer information on it.

The third finding is about the perception and attitude of cancer patients regarding CAM information from the media. We confirmed that cancer patients consistently pointed out the “lack of scientific basis or expert verification” of CAM information in the media. People wanted to get CAM information provided by experts, but they were not functioning properly as a cancer information acquisition channel. This result is similar to previous studies regarding CAM (13, 14). People’s demand for scientific-evidenced CAM information verified by experts has continued for over 10 years; however, the same situation continues without proper intervention from experts or governments. Cancer patients are measuring the effectiveness of cancer information by filtering information and conducting self-clinical explorations. The cancer patients filter information based on “common sense” or “feeling” without accurate evidence or knowledge, and such filtered information leads to self-medication. This can pose a serious health risk and lead to an increase in prevalence and cost. On several occasions, “true’ information is initially spread as rumors (24) because most people do not have the initial knowledge to evaluate it. This is especially true for medical information. The fact that cancer information belongs to the medical field is a factor that greatly discourages cancer patients who try to verify the accuracy of information through all available channels (42). For information regarded as “rumor” to function as “useful and reliable” information, verification and intervention by experts are essential. Experts or health authorities with relevant knowledge can be used as important news sources, providing evidence-based information when the uncertainty of the issue is high in the public perspective (43–45). For the information that has become a social issue, the government should have a systematic communication system that can promptly deliver evidence-based information from experts to the public. Notably, health authorities have distributed press releases before the celebrity’s article on taking fenbendazole, but this has not been communicated to the public. It is necessary to closely examine what caused the health authorities’ failure to communicate in the fenbendazole scandal. For this, we suggest that researchers examine how media reported their press releases, whether they were accurate and persuasive, and whether they were reported enough to reach the public. This is because the media should provide the content of the press release “sufficiently” while securing the quality of information so that the report is not misrepresented in health communication (44, 46). Moreover, we need to discuss the timing of the response. As people believe and spread false messages more than official messages when distrust of governments or official institutions increases (31), a preemptive response from the government and official institutions is urgently required before the anxiety and confusion of cancer patients increases. These additional studies and inductive analysis of the fenbendazole issue are expected to help prepare a rapid response system for false news that may occur in the future.

Finally, information that is far from the truth but seems true gets attention and spreads in the rumor-spreading model (32). The rationale for this reinforces factuality (which, herein, refers to how plausible a rumor or how specific information is presented) and becomes the driving force for the spread of rumors (47). Regarding CAM, the specific experiences of patients suffering from the same cancer operate with concrete information and evidence. The strong evidence of “experience” often overtakes patients’ common perception that CAM lacks scientific evidence (which is revealed in this study), leading them to accept and act on the information. As rumors that people perceive as reliable are spreading more (48), the perception of being “trustworthy” is created based on patients’ experiences (even if it is not scientific). Hopefully, the strong evidence of the patient’s experience will be countered by the active intervention of experts and the government. It is also necessary to introduce a program to improve patients’ cancer information literacy or a guideline on how to distinguish reliable information.

As everyone has become a producer and consumer of information, the freedom of publication and expression has been maximized, but cancer information should not be produced by everyone. Cancer is directly related to someone’s life and hence, evidence-based information that has been verified by experts needs to be provided and distributed. It must be accurate and reliable. Thus, discussions at the academic level for establishing a verification system for CAM information need to be conducted. For the sound distribution of accurate cancer information, some national intervention is also required. The intervention should be focused on effectively controlling and filtering false information. Specifically, at the national level, a system for monitoring and expert verification of information distributed to various media and platforms should be established, and the information needs of cancer patients should be continuously identified. Medical experts should actively and voluntarily participate in verifying information so that cancer patients are not threatened with wrong and false cancer information. The United States established the Office of Cancer Comprehensive Alternative Medicine (OCCAM) to continue CAM research and provide information, agreeing that its use has a positive effect on emotional relaxation or condition in cancer patients (3, 5, 6, 8). Through this, efforts are being made to present research data or evidence related to CAM. However, many countries do not have a research system or official institutions that provide scientific evidence-based CAM information. As is well known, many studies have shown that more than half of the world’s cancer patients use CAM (4, 5, 9, 11, 14). Health authorities should not neglect the reality that cancer patients miss the right timing of treatment by using incorrect CAM and it increases personal and social costs. It is urgent to change the perception of health authorities to establish an academic foundation for verifying the scientific evidence of CAM and to accumulate clinical experimental data and standardized protocols. Efforts to establish a cancer communication system at the national level for effective distribution of CAM information are also needed. Moreover, non-experts and cancer patients who have become active information providers through YouTube need to know the difference between sharing experiences, expressing their thoughts and beliefs, and providing evidence-based information. Preemptively, labeling that can distinguish between expert and non-expert information within platforms such as YouTube can be an alternative. For this, systematic communication and cooperation between medical experts, the government, and the media are essential. This is a massive, time-consuming task, but such efforts could build public trust in the health authorities. If the expert verification and communication system of health authorities is effectively operated in the early stages of the distribution of false health information, national and social confusion caused by false information can be prevented in advance. In addition, it goes without saying that public trust in health authorities will enable effective response to various health-related risk situations at the national level (eg., infectious diseases such as COVID-19). We have no reason to hesitate to build such a systematic communication system.

This study has some limitations. First, our data were collected only for Korean lung cancer patients. Although the results provide symbolic insight to enable a preemptive response to false cancer information by empirically showing its acquisition patterns among patients in complex media environments, the process can be affected by cultural differences. Referring to the results of this study, we recommend that future studies try to conduct an in-depth analysis of the process of obtaining false cancer information in other societies. Second, this study targeted lung cancer patients related to the use of fenbendazole, so the average age of respondents is higher and not diverse. Since this study focused on the empirical context of the false information acquisition process using qualitative research methods, it does not explain the general characteristics of cancer patients. This is according to the study’s purpose and method but examining the differences according to various characteristics of cancer patients, such as age, gender, and education, can greatly help in the segmentation strategy of cancer information users. Therefore, this study recommends that researchers try quantitative analysis to compare information acquisition patterns of various people. Quantitative analysis has some limitations in providing empirical and contextual analysis results, but it will allow assessment of the statistical significance of the analysis results and accumulate quantitative knowledge that can generalize factors that affect cancer patients’ information acquisition behavior. Finally, the results showed that at least network-based social media such as Facebook, Instagram, and Twitter were not involved in the process of acquiring false cancer information, but rather, the effect of mass media or YouTube, which could expose information to an unspecified number of people was strong. However, there is a possibility of a difference in the cancer information acquisition path between cancer that is mainly affected by relatively high age groups such as lung cancer and cancer that can affect young people. As the growth rate of platforms dealing with short content such as Instagram is remarkable recently, it would be a meaningful study to try to analyze if there is a case of false information distribution related to cancer, which is vulnerable to young people. The results of such research can provide an opportunity to empirically analyze whether there is a difference in the false information acquisition process according to age compared to this study’s results. Despite these limitations, this study, which examines the acquisition path of false cancer information according to the empirical context of cancer patients centering on an actual case in a complex media environment, would present meaningful results in the development of related research. It would present empirical evidence to help health authorities effectively and preemptively respond in the cancer communication context.

## Data availability statement

The raw data supporting the conclusions of this article will be made available by the authors, without undue reservation.

## Ethics statement

The studies involving human participants were reviewed and approved by The National Cancer Center Institutional Review Board in Korea (approval number: NCC2022-0001). Written informed consent for participation was not required for this study in accordance with the national legislation and the institutional requirements.

## Author contributions

JK, HS, KO, and JJ certify that they contributed substantially to the conception, design, and analysis of the paper, and the authors participated in drafting and revising the manuscript. All authors contributed to the article and approved the submitted version.

## Funding

This study was supported by a Grant-in-Aid from the National Health Promotion Fund (grant number 2260010-1), Ministry of Health and Welfare, Republic of Korea. Funding bodies have no role in the study design, study setting, analysis, or writing of the manuscript.

## Conflict of interest

The authors declare that the research was conducted in the absence of any commercial or financial relationships that could be construed as a potential conflict of interest.

## Publisher’s note

All claims expressed in this article are solely those of the authors and do not necessarily represent those of their affiliated organizations, or those of the publisher, the editors and the reviewers. Any product that may be evaluated in this article, or claim that may be made by its manufacturer, is not guaranteed or endorsed by the publisher.
